# 骨代谢标志物ICTP、BAP对肺癌骨转移的诊断意义

**DOI:** 10.3779/j.issn.1009-3419.2010.10.04

**Published:** 2010-10-20

**Authors:** 宇 忻, 宝惠 韩, 家陶 娄, 京 吴, 艳洁 牛

**Affiliations:** 1 200030 上海，上海交通大学附属胸科医院肺内科 Department of Pulmonary Medicine, Shanghai Chest Hospital, Shanghai Jiaotong University, Shanghai 200030, China; 2 200030 上海，上海交通大学附属胸科医院检验科 Department of Laboratory Medicine, Shanghai Chest Hospital, Shanghai Jiaotong University, Shanghai 200030, China

**Keywords:** 肺肿瘤, 骨转移, ICTP, BAP, 酶联免疫法, 电化学发光免疫分析法, Lung neoplasms, Bone metastases, ICTP, BAP, Enzyme-linked immunoassay, Electrochemiluminescence immunoassay

## Abstract

**背景与目的:**

骨代谢标志物是一类源于骨基质或骨细胞的代谢指标，可反映骨代谢情况。本研究旨在探讨血清骨代谢生化指标Ⅰ型胶原交联羧基端肽（cross-linked telopeptide of type Ⅰ collage, ICTP）和骨特异性碱性磷酸酶（bone-specific alkaline phosphatase, BAP）在肺癌骨转移诊断中的意义及其临床应用价值。

**方法:**

采用前瞻性对照方法，共入组110例，分为3组。初治肺癌患者共90例，临床分期为Ⅳ期，分为骨转移组50例和非骨转移组40例，健康对照组20例，采用酶联免疫法和电化学发光免疫测定法检测所有入组者治疗前的血清骨代谢生化指标ICTP和BAP水平，对骨转移的各因素与血清ICTP和BAP的相关性进行统计学分析。

**结果:**

骨转移组的血清ICTP和BAP水平显著高于非骨转移组和健康对照组（*P* < 0.05）。多发性骨转移组（骨转移数≥3）的血清BAP水平显著高于少发性骨转移组（骨转移数 < 3）（*P* < 0.05）。混合性骨转移组的血清BAP水平显著高于溶骨性骨转移组（*P* < 0.05）。ICTP和BAP在肺癌骨转移诊断中的敏感性分别为18%和40%，特异性分别为98.3%和95%，准确度分别为61.8%和70%。联合检测血清ICTP和BAP可提高其敏感性和准确度（分别为52%和74.5%）。

**结论:**

由于检测方便、无创性、成本较低，血清骨代谢生化指标ICTP和BAP在肺癌骨转移的临床诊断中有一定的实用价值。

## Abstract (en)

**Background and objective:**

Bone metabolic markers are biomarkers derived from bone matrix or osteocyte reflecting bone metabolic situation. The aim of this study is to explore the diagnostic value of bone markers, cross-linked telopeptide of type Ⅰ collage (ICTP) and bone-specific alkaline phosphatase (BAP) in lung cancer patients with bone metastases (B.M).

**Methods:**

As a prospective control study, 110 cases were enrolled and divided into 3 groups. Totally 90 pre-treated, stage Ⅳ lung cancer cases were divided into two groups: 50 cases with bone metastases, 40 cases without bone metastases, as well as 20 healthy subjects were included as control. Serum ICTP was detected by enzyme-linked immunoassay (ELISA) and serum BAP was detected by electrochemiluminescence immunoassay. The relationships between factors of B.M and serum levels of ICTP, BAP were analyzed.

**Results:**

The levels of serum ICTP and BAP in lung cancer patients with B.M were significantly higher than those in the healthy controls and those without B.M (*P* < 0.05). The level of serum BAP in patients with multiple B.M (lesions≥3) was significantly higher than that in patients with few B.M (lesions < 3) (*P* < 0.05). The level of serum BAP in patients with mixed pattern was significantly higher than that with lytic pattern. The sensibility of ICTP and BAP in the diagnosis of B.M in lung cancer patients was 18% and 40% respectively. The specificity of ICTP and BAP was 98.3% and 95% respectively. The accuracy rates of ICTP and BAP were 61.8% and 70.0% respectively. Joint detection of ICTP and BAP had improved sensibility and accuracy in the diagnosis of bone metastases in lung cancer patients (52% and 74.5% respectively).

**Conclusion:**

Serum bone biochemical markers ICTP and BAP have diagnostic values for B.M in lung cancer patients with lower costs, noninvasiveness and convenience.

肺癌骨转移常导致严重的骨骼病变，包括骨疼痛、病理性骨折、脊髓压迫、高钙血症等骨相关事件（skeletal related event, SRE），不仅严重影响了患者的生活质量，而且威胁患者的生存。因而如何尽早正确诊断肺癌骨转移、进行早期干预以消除转移灶或减缓转移灶的发展，对提高患者的生活质量和延长生存期均有着非常重要的现实意义。

近年来骨代谢生化标志物的检测发展很快，可以迅速、全面地反映骨代谢情况。本研究通过酶联免疫法和电化学发光免疫分析法测定血清骨代谢标志物Ⅰ型胶原交联羧基端肽（cross-linked telopeptide of type Ⅰ collage, ICTP）和骨特异性碱性磷酸酶（bone-specific alkaline phosphatase, BAP）水平，旨在探讨血清ICTP和BAP在肺癌骨转移中的诊断价值，寻求传统的诊断骨转移方法之外的另一种新的更便捷的骨转移诊断的辅助方法。

## 1 材料与方法

### 1.1 研究对象

本研究为前瞻性对照研究，经知情同意后入组，共入组110例，肺癌患者共90例，健康对照组20例。肺癌患者为2009年7月-2010年2月上海交通大学附属胸科医院住院患者，均经病理学或细胞学证实为原发性支气管肺癌，根据国际抗癌联盟UICC 2009年制定的新TNM分期，临床分期为Ⅳ期的初治患者。分为骨转移组和非骨转移组。骨转移组50例，其中男37例，女13例，年龄35岁-76岁，中位年龄57.5岁。非骨转移组40例，经临床检查和同位素骨扫描排除骨转移，存在骨外器官转移。其中男30例，女10例，年龄31岁-77岁，中位年龄59岁。健康对照组20例，其中男15例，女5例，年龄25岁-62岁，中位年龄45岁。在入组前一年内均无外伤性骨折、风湿性或骨关节炎及临床或X线有明确的骨质疏松症，近3个月内未使用影响骨代谢药物，如维生素D、雌激素、钙制剂、双磷酸盐、氟制剂以及糖皮质激素。排除甲亢、甲旁亢、糖尿病、皮质醇增多症等所致继发性骨质疏松症，无严重心、肝、肾疾病。

### 1.2 诊断方法

肺癌患者行体检、胸部CT、脑CT、腹部B超、纤维支气管镜、骨同位素扫描或PET检查，确定临床分期为Ⅳ期的肺癌患者。骨转移的诊断标准为：①骨穿刺活检证实为转移性骨肿瘤；②骨同位素扫描示3处或3处以上散在异常放射性核素分布（核素浓聚灶或减低灶），骨转移症状明显，且排除外伤及其它骨骼病变；③骨同位素扫描示单处及2处放射性分布异常者，经X线或CT、MRI检查提示骨转移；④放射性核素检查阴性，但临床有骨转移症状，X线或CT、MRI检查提示骨转移。所有入组者均行血清ICTP、BAP检测。

### 1.3 检测方法

#### 1.3.1 标本采集

所有入组者抽取晨间空腹静脉血2 mL，离心分离血清，分装于-80 ℃冰箱保存。

#### 1.3.2 血清ICTP的检测方法

仪器设备：安图2010酶标仪。采用试剂为ICTP试剂盒（美国ADL公司）。实验方法：酶联免疫法。结果判断：仪器值：在波长450 nm的酶标仪上读取各孔的OD值；B=标准品OD值，B0=标准品0点OD值，以B/B0%值为纵坐标，以标准品浓度为横坐标，在对数坐标纸上绘制标准曲线，在标准曲线上查找对应的浓度范围；敏感度：0.01 ng/mL。

#### 1.3.3 血清BAP的检测方法

仪器设备：UNICEL DXI 800化学发光仪，采用Access BAP试剂盒（Beckman CounLter公司），实验方法：一步法酶免疫分析。结果判断：患者的检测结果由仪器软件自动通过平滑的三次方的参数曲线计算方式进行统计，样品中检测到的光量子值会与储存的标准曲线对应求得相应结果。

### 1.4 统计学方法

运用SPSS 13.0软件包进行统计分析。正态分布检验采用*One-Sample Kolmogorov-Smirnov* Test，两样本均数比较采用*Independent Samples* Test的*t*检验，非参数检验采用*Mann-Whitney* Test，*P* < 0.05为差异有统计学意义。

## 2 结果

### 2.1 患者基本情况

非骨转移组转移部位分别为胸膜15例（37.5%），肺13例（32.5%），脑8例（20%），肾上腺2例（5%），肝脏1例（2.5%），胰腺1例（2.5%）。肺癌骨转移组及非骨转移组临床资料见表 1。

**1:** 90例Ⅳ期原发性支气管肺癌患者临床资料 Clinical data of 90 stage Ⅳ lung cancer patients

Clinical characteristics	Bone metastases (*n*=50)	Without bone metastases (*n*=40)
Gender		
Male	37	30
Female	13	10
Age (yrs)	35-76	31-77
Median age (yrs)	57.5	59.0
Histological type (n, %)		
Adenocarcinoma	36 (72%)	22 (55%)
Squamous cell carcinoma	5 (10%)	8 (20%)
Squamous-adeno	1 (2%)	1 (2.5%)
Small cell	5 (10%)	5 (12.5%)
Large cell	1 (2%)	0
Others	2 (4%)	4 (10%)
Gross type		
Central type	20 (40%)	17 (42.5%)
Peripheral type	30 (60%)	23 (57.5%)
T stage		
T1	2	0
T2	30	25
T3	2	3
T4	16	12
N stage		
N0	1	0
N1	3	1
N2	25	20
N3	21	19
PS		
0	4	3
1	38	33
2	7	4
3	1	0

### 2.2 骨转移患者临床病理特点

骨转移患者50例，2例经骨穿刺活检病理证实为转移性骨肿瘤。根据同位素骨扫描表现，26例为多发性骨转移（骨转移数≥3），24例为单发或2个放射性浓聚灶，经进一步行X线、CT、MRI或18FPET证实为骨转移。

由两名经验丰富的放射科医师进行X线、CT、MRI及PET-CT读片，根据影像学分型，溶骨性40例，成骨性5例，混合性5例。

骨转移伴有其它脏器转移者30例，转移部位有肺（11例）、肝（11例）、脑（7例）、胸膜（6例）、腹腔淋巴结（3例）、肾上腺（2例）、皮下（2例）、胰腺（1例）、肾（1例）、心包（1例）。单纯骨转移者20例。

为了便于分析肺癌骨转移的分布特点，参照WiLson的做法^[7]^，将人体骨骼分为5个区（包括15个部位）：①胸部（30例，60%）：肋骨、锁骨、胸骨、肩胛骨；②脊柱（32例，64%）：颈椎、胸椎和腰椎；③骨盆（22例，44%）：髂骨、坐骨、耻骨、骶骨及骶髂区；④肢体（24例，48%）：上肢和下肢；⑤颅骨（2例，4%）。疼痛评分情况：本组骨转移患者26例无疼痛症状，24例患者存在不同程度的疼痛，根据WHO简易疼痛强度分级法（VRS）分级，1级10例，2级11例，3级3例。多数患者的疼痛表现为胸痛，其次为腰背部疼痛，其它依次为骨盆、肢体、颅骨等部位的疼痛。

贫血情况：骨转移组治疗前存在不同程度的贫血有8例（16%），其中血红蛋白值< 100 g/L的2例均为多发骨转移（骨转移数≥3）。

骨相关事件：共8例。5例病理性骨折。1例为胸腰椎转移引起的脊髓压迫症状而行放疗。2例为背部重度疼痛经MRI证实为胸椎骨质破坏而行放疗。

### 2.3 各组血清ICTP和BAP检测结果的比较

各组血清ICTP和BAP检测结果的比较见表 2、表 3。

**2:** 各组血清ICTP检测结果的比较（Mean±SD） Comparison of mean values of serum ICTP in groups (Mean±SD)

Group	*n*	ICTP (*μ*g/L)	*P*
^*^Bone metastases	50	2.71±1.39	0.001^*★^
^★^Without bone metastases	40	1.85±0.84	0.523^★▲^
^▲^Healthy controls	20	2.00±0.87	0.036^*▲^
^*★^Comparison of mean values of serum ICTP in bone metastases group and without bone metastases group (*P*=0.001); ^*▲^bone metastases group and healthy controls (*P*=0.036); ^★▲^There was no statistically significant relationship between without bone metastases group and healthy controls (*P*=0.523). ICTP: cross-linked telopeptide of type Ⅰ collage; BAP: bone-specific alkaline phosphatase.			

**3:** 各组血清BAP检测结果的比较（Median） Comparison of median of values of serum BAP in groups (Median)

Group	*n*	BAP (*μ*g/L) [Median (range)]	*P*
^*^Bone metastases	50	16.70 (7.48-109.00)	< 0.001^*★^
^★^Without bone metastases	40	11.40 (5.54-28.40)	0.130^★▲^
^▲^Healthy controls	20	9.89 (5.49-15.80)	< 0.001^*▲^
The detection results of serum BAP were expressed by Median. ^*★^Comparison of serum BAP in bone metastases group and without bone metastases group (*P* < 0.001); ^*▲^bone metastases group and healthy controls (*P* < 0.001); ^★▲^There was no statistically significant relationship between without bone metastases group and healthy controls (*P*=0.130).			

### 2.4 肺癌骨转移组的分层研究

50例骨转移组患者分层研究结果见表 4，ICTP结果以Mean±SD表示，BAP结果以中位数表示。血清ICTP、BAP水平与骨转移组年龄、性别、病理类型、T分期、N分期、骨转移是否伴有其它脏器转移、有无疼痛、贫血、骨相关事件无显著相关（*P* > 0.05）。多发性骨转移组（骨转移数≥3）的血清BAP水平显著高于少发性骨转移组（骨转移数 < 3）（*P* < 0.05）。混合性骨转移组的血清BAP水平显著高于溶骨性骨转移组（*P* < 0.05）。多发性骨转移组与少发性骨转移组间、不同影像学类型间血清ICTP水平差别无统计学意义（*P* > 0.05）（表 4）。

**4:** 肺癌骨转移患者分层因素血清ICTP和BAP水平的比较 Comparisons of serum ICTP and BAP in bone metatases patients with demixing factors

Factors	*n*	ICTP (μg/L) (Mean±SD)	*P*	BAP (*μ*g/L) (Median)	*P*^#^
Age (yrs)			0.731		0.107
≥60	20	2.80±1.14		20.60	
< 60	30	2.66±1.55		13.90	
Gender			0.604		0.543
Male	37	2.77±1.52		15.70	
Female	13	2.54±0.97		21.10	
Metastases status			0.253		0.851
Bone metastases only	20	2.99±1.80		16.60	
With other distant metastases	30	2.53±1.02		18.45	
Site involvement			0.825		0.009
Few-site involvement (< 3)	24	2.67±1.74		13.90	
Multiple-site involvement (≥3)	26	2.76±1.00		25.05	
Pain status			0.597		0.846
With pain	24	2.83±1.17		18.20	
Without pain	26	2.61±1.58		15.45	
Anemia status			0.603		0.397
With anemia	8	2.95±1.01		21.40	
Without anemia		2.67±1.46		15.35	
SREs			0.528		0.234
With SREs	8	2.43±1.22		13.55	
Without SREs	42	2.77±1.43		17.60	
Imaging patterns					
Lytic^*^	40	2.79±1.45^*^	0.135^*★^	14.90^*^	0.367^*★^
Blastic^★^	5	1.76±1.15^★^	0.065^★▲^	26.80^★^	0.076^★▲^
Mixed^▲^	5	3.08±0.77^▲^	0.659^*▲^	73.10^▲^	0.001^*▲^
Histological type			0.137		0.130
Adenocarcinoma	36	2.90±0.77		21.05	
Others	14	2.24±0.92		13.90	
T stage			0.750		0.196
T1+T2	32	2.76±1.57		20.60	
T3+T4	18	2.63±0.01		14.20	
N stage			0.493		0.969
N0+N1+N2	29	2.83±1.52		17.50	
N3	21	2.55±1.21		15.90	
^#^Comparisons of level of BAP in each group were analyzed by *Mann*-*Whitney* Test, expressed by Median. ^*^Lytic; ^★^Blastic; ^▲^Mixed.					

### 2.5 血清ICTP和BAP单检及联合检测的诊断评价指标

血清ICTP以 > 3.5 μg/L为阳性。BAP男性正常值范围0 μg/ L-20.10 μg/L，以 > 20.10 μg/L为阳性，女性正常值绝经前为0 μg/L-14.3 μg/L，绝经后为0 μg/L-22.4 μg/L，正常值范围标准参照试剂盒说明书，以大于正常值上限为阳性。

血清ICTP和BAP单检及联合检测的诊断评价指标见表 5。

**5:** 血清ICTP和BAP单检及联合检测的诊断评价指标 Diagnostic values in detections of serum ICTP and BAP respectively and combined detection

Item	Sensibility (%)	Specificity (%)	Accuracy (%)	Positive predicted value (%)	Negative predicted value (%)
ICTP	18.0 (9/50)	98.3 (59/60)	61.8 (68/110)	90.0 (9/10)	59.0 (59/100)
BAP	40.0 (20/50)	95.0 (57/60)	70.0 (77/110)	87.0 (20/23)	65.5 (57/87)
ICTP+BAP	52.0 (26/50)	93.3 (56/60)	74.5 (82/110)	86.7 (26/30)	70.0 (56/80)

血清ICTP的敏感性为18%（9/50），特异性为98.3%（59/60），准确度为61.8%（68/110）。血清BAP的敏感性40%（20/50），特异性为95.0%（57/60），准确度为70.0%（70/110）。血清ICTP和BAP联合检测诊断的敏感性为52.0%（26/50），特异性为93.3%（56/60），准确度为74.5%（82/110）。

## 3 讨论

目前越来越多的研究显示骨代谢相关的生化指标可成为肿瘤骨转移潜在的预测、诊断和预后因素。骨代谢生化指标按其所处过程分为两类，即骨形成指标和骨吸收指标。骨中的骨基质为成骨细胞分泌，其内的有机质90%-95%为骨胶原。其中绝大多数为Ⅰ型胶原，极少数为Ⅴ型胶原。骨髓系统有转移病变时首先影响到胶原蛋白的代谢变化。近来，一些新的生化标志物出现，它们是Ⅰ型胶原的分解产物，由于骨转移的累及范围及活动程度与骨代谢标志物之间存在一定的相关性，故通过骨代谢标志物的变化可以评估治疗的疗效以及疾病的转归。

Ⅰ型胶原蛋白通过基质金属蛋白酶-9（matrix metallopeptidase 9, MMP-9）介导，在病理性骨骼降解过程中生成ICTP。ICTP参与三价交叉联合，血液中可见这种终肽的免疫生化完整形式，它衍生于骨骼的重吸收和疏松结构组织的降解，反映骨吸收，其升高程度与破骨细胞活性的增高是一致的^[1]^。ICTP以完整的免疫源性肽形式进入血液，不再进一步分解，并不受摄入食物的影响。有研究^[2]^认为不论何种类型的肿瘤，Ⅰ型胶原的降解产物是鉴别患者有无骨转移的最好的指标。

通过抗体识别Ⅰ型胶原α1链上独特序列中包含的两个苯基丙氨酸可检测血清中ICTP的浓度。本研究显示肺癌骨转移组血清ICTP的均值较肺癌非骨转移组、健康对照组均有显著增高（*P* < 0.05），显示血清ICTP水平对肺癌骨转移有一定的诊断价值。研究显示在肺癌骨转移中ICTP是一个诊断疾病进展和预后的指标。Horignchi等^[3]^发现肺癌骨转移患者的血清ICTP水平比肺癌无骨转移和肺良性疾病的患者高，并且其水平与骨转移的程度呈正相关。且ICTP不受月经状态影响，与骨累及程度相关性最好。Nakamura等^[4]^比较了NTx和ICTP的在肿瘤骨转移中的诊断价值，发现两者均明显升高。有研究^[5]^表明ICTP和NTx与肿瘤骨转移疾病的进展有显著相关。Martinetti等^[6]^在对伴有骨痛的乳腺癌、甲状腺癌等骨转移患者的一系列骨代谢标志物的研究中发现ICTP是唯一与癌症骨痛相关的标志物。而本研究未发现骨痛与血清ICTP水平的相关性（*P* > 0.05）。

本研究显示骨转移组年龄、性别、骨转移数（≥3和 < 3）、T分期、N分期与血清ICTP水平无显著相关。骨转移伴有其它脏器转移与单骨转移、腺癌与非腺癌血清ICTP水平无显著差异。血清ICTP水平在溶骨性、成骨性和混合性三组间差别无统计学意义。

血清BAP由成骨细胞分泌，其生理功用主要是在成骨过程中水解磷酸酯和焦磷酸盐，在骨矿化过程中增加局部无机磷的浓度，有利于成骨过程。BAP水平与成骨细胞和前成骨细胞活性呈线性相关，被认为是最精确的骨形成标志物。BA P活性反映了骨转换速率，一些文献^[7]^研究发现BAP与各部位的骨密度呈负相关，是公认的骨质疏松症疗效的评价指标，用于骨质疏松症的筛查、辅助诊断和疗效观察。BAP在实体瘤骨转移方面的价值也已得到充分肯定。Pectasides^[8]^在乳腺癌骨转移的研究中发现由于肿瘤骨转移破坏了骨重建过程的平衡，未经治疗的骨转移患者中的血清BAP水平显著升高，并且ROC曲线分析显示在乳腺癌骨转移中BAP比NTx诊断准确度更高。有研究^[9]^表明血清BAP水平对男性肺癌骨转移有诊断价值。

BAP作为成骨细胞的一种成分参与成骨过程，其活性在血中稳定，没有昼夜变化^[10]^。应用单克隆抗体建立的免疫分析法具有高度敏感性和特异性、重复性好、操作简便等特点，是目前鉴定和定量骨特异性碱性磷酸酶最普遍和最佳的方法。

本研究显示肺癌骨转移组血清BAP水平显著高于肺癌非骨转移组和健康对照组（*P* < 0.05），血清BAP诊断的敏感性为40%，特异性为95.0%，准确度为70.0%，表明血清BAP对肺癌骨转移有一定的诊断价值。文献^[11]^报道BAP在乳腺癌骨转移的诊断中有较高的准确性，其敏感性为40%-80%，特异性为75%-90%。Marcher等^[12]^发现在骨扫描阳性前数月BAP即有升高。

在本组骨转移组的分层研究中，骨转移数≥3患者的血清BAP水平显著高于骨转移数 < 3，两组比较差别有统计学意义（*P* < 0.05）。显示血清BAP水平与骨转移的范围和程度相关，与Pectasides^[8]^在乳腺癌骨转移中的研究报道一致。Berruti等^[13]^通过对323例骨转移患者的研究发现BAP与成骨性骨转移的累及范围相关。

本研究显示混合性、成骨性两组中的血清BAP水平要高于其在溶骨性组中的水平，混合性组与溶骨性组的血清BAP水平差别有统计学意义（*P* < 0.05），进一步分析示5例混合性骨转移中4例为全身广泛骨转移，1例为2处转移，其血清BAP水平显著升高。成骨性组中2例为单发骨转移，故BAP水平与骨转移累及的范围和程度有关。本组成骨性组和混合性组的样本较小，需在今后的研究中扩大样本量来统计。

本研究显示骨转移组年龄、性别、T分期、N分期与血清BAP水平无显著相关。骨转移伴有其它脏器转移与单骨转移间、腺癌与非腺癌血清间BAP水平无显著差异。血清BAP水平与患者是否伴有疼痛无相关性。

目前用于骨转移的诊断和随访的方法主要还是影像学检查包括X线平片、CT、MRI、PET和同位素骨扫描。然而每项影像学检查都有其局限性，如PET检查价格昂贵，并且由于患者对于放射性损伤的惧怕而限制了在骨转移治疗随访中经常重复这些检查。在骨转移的诊断和随访中骨生化指标在以下几个方面优于影像学检查：①骨生化指标有提高其敏感性的潜力；②骨生化指标与系统性的事件相关而不是仅反映局部事件；③骨生化指标对治疗的反应更快捷；④指标可鉴别骨转移灶的治愈或进展；⑤骨生化指标可在骨损害的细胞学的机制上提供更多的信息^[14]^。

目前关于骨代谢生化指标的大量研究主要集中在乳腺癌和前列腺癌骨转移上。本研究结果血清ICTP和BAP的诊断敏感性分别为18%和40%，诊断的特异性分别为98.3%和95%。联合检测两项指标可提高其敏感性（52%）和准确度（74.5%），特异性为93.3%，因此我们认为联合检测血清ICTP、BAP对骨转移的诊断有一定的价值。同位素骨扫描诊断敏感性较高，特异性较低，而血清ICTP、BAP敏感性低，特异性高，因此肺癌患者行同位素骨扫描检查可与检测血清ICTP、BAP结合，以期提高对患者骨转移诊断的准确度，这方面有待进一步的研究探讨。

虽然目前这两项指标的检测在肺癌骨转移的诊断或筛查上不能取代同位素骨扫描及其它影像学检查，但由于其检测方便、无创性、成本较低，便于在肺癌骨转移的治疗及随访过程中进行复查，可能成为评价骨转移治疗疗效的一种方法。
